# Derivation of Fiber Orientations From Oblique Views Through Human Brain Sections in 3D-Polarized Light Imaging

**DOI:** 10.3389/fnana.2018.00075

**Published:** 2018-09-27

**Authors:** Daniel Schmitz, Sascha E. A. Muenzing, Martin Schober, Nicole Schubert, Martina Minnerop, Thomas Lippert, Katrin Amunts, Markus Axer

**Affiliations:** ^1^Institute of Neuroscience and Medicine-1 (INM-1), Forschungszentrum Jülich, Jülich, Germany; ^2^>Center for Movement Disorders and Neuromodulation, Department of Neurology and Institute of Clinical Neuroscience and Medical Psychology, Medical Faculty, Heinrich-Heine University, Düsseldorf, Germany; ^3^Jülich Supercomputing Center, Forschungszentrum Jülich, Jülich, Germany; ^4^Bergische Universität Wuppertal, Wuppertal, Germany; ^5^C. and O. Vogt Institute for Brain Research, Medical Faculty, Heinrich-Heine University Düsseldorf, Düsseldorf, Germany

**Keywords:** neuroimaging, modeling, 3D-PLI, white matter anatomy, fiber architecture

## Abstract

3D-Polarized Light Imaging (3D-PLI) enables high-resolution three-dimensional mapping of the nerve fiber architecture in unstained histological brain sections based on the intrinsic birefringence of myelinated nerve fibers. The interpretation of the measured birefringent signals comes with conjointly measured information about the local fiber birefringence strength and the fiber orientation. In this study, we present a novel approach to disentangle both parameters from each other based on a weighted least squares routine (ROFL) applied to oblique polarimetric 3D-PLI measurements. This approach was compared to a previously described analytical method on simulated and experimental data obtained from a post mortem human brain. Analysis of the simulations revealed in case of ROFL a distinctly increased level of confidence to determine steep and flat fiber orientations with respect to the brain sectioning plane. Based on analysis of histological sections of a human brain dataset, it was demonstrated that ROFL provides a coherent characterization of cortical, subcortical, and white matter regions in terms of fiber orientation and birefringence strength, within and across sections. Oblique measurements combined with ROFL analysis opens up new ways to determine physical brain tissue properties by means of 3D-PLI microscopy.

## 1. Introduction

Understanding the human brain's function and dysfunction requires a thorough knowledge about the brain's fiber tracts, forming a dense network of connections within, but also between the different brain regions. Over the last years, several imaging techniques have emerged which are capable of resolving anatomical structures with different spatial resolutions. At the millimeter scale, diffusion MRI is the most prominent one as it is applicable to both *in vivo* and post mortem brains (Basser et al., 1994; Johansen-Berg and Behrens, 2009; Mori and Tournier, 2014). At ultra-high resolution two-photon microscopy (Laperchia et al., 2013), light-sheet microscopy (Silvestri et al., 2012), and electron microscopy (Knott et al., 2008), amongst others, have been exploited to image single cells and neurons in 3D space as well as their local connections. Yet these techniques come with the cost of excessive measurement time, large amounts of data and limited fields of view (lateral and axial), impeding the study of larger brain volumes so far.

3D-Polarized Light Imaging (3D-PLI) (Axer et al., 2011a,b) is a microscopic technique that bridges the gap between large-scale imaging techniques such as diffusion MRI and ultra-high resolution microscopy. It enables the reconstruction of fiber tracts at the meso- and micro-scale from unstained histologial sections. Recently, polarization sensitive optical coherence tomography (PSOCT) has emerged as a promising technique for the mapping of fiber bundles with the ability of depth-resolved scanning of brain blocks (Wang et al., 2011, 2014; Magnain et al., 2014). While the signals measured by PSOCT and 3D-PLI both arise due to the birefringence of brain tissue, their fundamental difference is that PSOCT captures the reflected light instead of the transmitted light as 3D-PLI (Caspers and Axer, 2017).

A pitfall of any histological imaging technique is the extraction of information in the direction perpendicular to the sectioning plane, i.e., in the depths of the histological section. While in-plane fiber orientations can be obtained from texture information of microscopic images via the structure tensor without the need for any biophysical model (Budde and Frank, 2012), little information is directly available about orientations perpendicular to the sectioning plane. Lately, three-dimensional structure analysis has been applied to image stacks obtained from light microscopy (Khan et al., 2015) and confocal microscopy (Schilling et al., 2016). This comes with several difficulties: the prerequisites of a precise 3D-alignment of the image stack as well as a coherent contrast over the whole image stack and the strong dependency of the obtained orientations on the chosen pre-processing pipeline and kernel size of the structure tensor (Khan et al., 2015).

As an alternative, 3D-PLI enables to determine fiber orientations directly from the measurement data without the need for a manual choice of image processing parameters. This is achieved by taking measurement data from different views by tilting the brain section and analyzing it with a biophysical model (Axer et al., 2011a). Especially, the estimation of the out-of-sectioning-plane orientation (inclination angle) benefits from the additional information gained by tilting the brain section as it is measured conjointly with the fiber density.

Several algorithms for the analysis of the data acquired with the tiltable specimen stage have been presented so far. The Markov Random Field algorithm by Kleiner et al. (2012) and the total variation-cased method by Alimi et al. (2017) both resulted in a robust estimation of the inclination sign. While this solved the inclination sign ambiguity, the absolute inclination was still undetermined. This problem was first solved by Wiese et al. (2014) who showed how the tilted measurements can be utilized to extract inclination and fiber density independently from each other. Their algorithm is based on a discrete Fourier transformation of the different tilting positions (denoted as DFT algorithm). Due to its analytical nature, the DFT algorithm is computationally efficient but suffers from noise instability.

The here presented approach seeks to overcome this noise instability and provide a more robust separation of fiber density and orientation. Our approach utilizes a weighted least squares algorithm to process the tilted measurements. It is then compared to the analytical DFT algorithm on synthetic and experimental data. The examined experimental results represent the first analysis of large-scale 3D-reconstructed human brain data in 3D-PLI. The new approach was introduced in Schmitz et al. (2018) for the first time, yet this work represents a vast extension of the analysis and results.

## 2. Materials and methods

### 2.1. Least squares estimation of fiber parameters

#### 2.1.1. 3D-PLI

**Figure 1:**
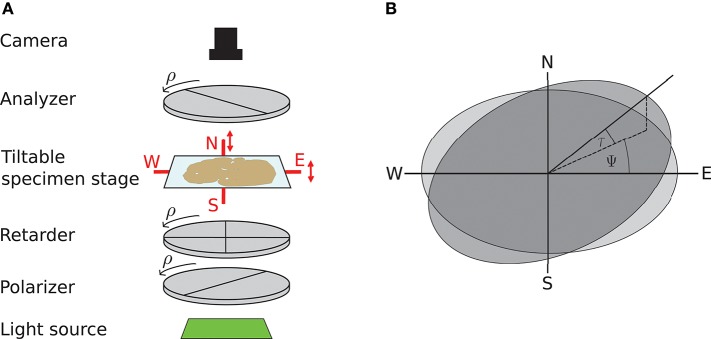
Experimental setup and tilting coordinate system. **(A)** Experimental setup. ρ denotes the rotation angle of the polarization filters. Red arrows depict possibility to tilt the specimen stage. **(B)** Tilting coordinate system. Light gray: specimen stage in the planar position without tilt, dark gray: tilted specimen stage. ψ, tilting direction angle; τ, tilting angle. Image courtesy: N. Gales.

3D-PLI utilizes the birefringence of nerve fibers which is measured in customized polarimeters. The birefringence originates from the regular arrangement of lipids in the myelin sheath resulting in optical anisotropy. This anisotropy causes a phase shift of incident polarized light passing the brain tissue. The optical setup as described in Axer et al. (2011b) is depicted in Figure 1: first unpolarized light from an LED array (custom-made design, FZJ-SSQ300-ALK-G, iiM, Germany) passes a first polarization filter and a quarter-wave retarder mounted with a principle axis offset of 45° with respect to the polarizer (Jos. Schneider Optische Werke GmbH, Germany), circularly polarizing the light. Then the light interacts with the brain tissue mounted on a tilting stage. A second polarizer, rotated 90° with respect to the first one serves as analyzer. The outgoing light is captured with a CCD camera with 14 bit depth (AxioCam HRc, Zeiss, Germany), capturing images at a pixel size of 64 × 64μm. The filters and the retarder are rotated simultaneously in steps of ρ = 10°, yielding an image series acquired by the camera.

The effective physical model behind 3D-PLI describes myelinated nerve fibers as negative uniaxial birefringent crystals. Assuming ideal optical components, this model yields the following expression of the light intensity in each pixel during rotation of the filters (Axer et al., 2011a):

(1) $$\begin{array}{l} I\left(\rho ,\varphi ,\delta \right)=\frac{I_{T}}{2}\cdot \left(1+\sin\left(2\left(\rho -\varphi \right)\right)\cdot \sin\delta \right) \end{array}$$

with the *transmittance* $\frac{I_{T}}{2}$, the in-plane orientation φ (*direction angle*), and the *retardation* δ. For the retardation the following expression is presumed:

(2) $$\begin{array}{l} \delta =\frac{\pi }{2}d\cos^{2}\left(\alpha \right) \end{array}$$

*d* with the *relative thickness* *d* and the out-of-plane orientation α (*inclination angle*). For readability purposes we denote *d* as *t*_*rel*_ in contrast to Axer et al. (2011a). The angles are defined in the range φ ∈ [0°, 180°] and α ∈ [−90°, 90°]. *d* was introduced by Axer et al. (2011a) as the combined effect of section thickness *t*_*s*_, birefringence Δ*n*, and illumination wavelength λ:

(3) $$\begin{array}{l} d=4\frac{t_{s}\Delta n}{\lambda } \end{array}$$

The sinusoidal profile is analyzed with a Fourier analysis: the *I*_*T*_ is given by the average of the profile, the direction angle by its phase and the retardation sinδ by its relative amplitude (Axer et al., 2011a). While this analysis enables unambiguous determination of transmittance, direction angle, and retardation, α and *d* are mapped onto one value, the retardation. Disentanglement of both parameters is a priori not possible, as any combination of *d* and α which fulfills Equation (2) is valid solution. Furthermore, for *d* > 1 the outer sin function induces an additional ambiguity. Therefore, the separation of *d* and α is only bijective for *d* ∈ (0, 1]. In Axer et al. (2011a) a constant relative thickness which was extracted by statistical approach is assumed. This enables the calculation of the inclination by inverting Equation (2). While this approach offers a good first guess for the inclinations, the assumption of a constant *d* over the whole section did not take local differences between distinct brain regions into account. For example, the cortex contains far less myelinated axons than dense white matter regions such as the corpus callosum. Also, it has to be noted that the relative thickness depends on the section thickness which is not precisely known and the birefringence which depends on the local amount of myelin (Goethlin, 1913). Therefore, it is clear that the unbiased disentanglement of both parameters requires additional measurement information.

A mechanical solution to gain more measurement information is a tiltable specimen stage built into the polarimetric system, which introduces a pre-defined tilting angle to the nerve fiber orientation relative to the sectioning plane (Axer et al., 2011a). By this means, polarimetric measurements can be performed from oblique views. Experimentally, this is realized by a two-nested axis system enabling rotations of the specimen around the x- and y-axis by up to 8°. In routine 4 tilting positions are measured: at each position the brain section is tilted by ±8° with respect to one of the axes, denoted as N(orth), S(outh), E(ast), and W(est) (see Figure 1). All measurements taken with a tilted sample are registered on the measurement without tilt (*planar measurement*) by a projective linear transformation.

#### 2.1.2. Light intensity distribution of the imaging system

For an accurate model of the data acquisition, the noise level of the imaging system must be taken into account. As the detected signals are light intensities captured by a CCD camera in our case, the dominant noise sources are shot noise during image acquisition and the internal signal processing of the camera (Bertolotti, 1974; Goodman, 2000). Here, the distribution of interest is the distribution of the number of detected photoelectrons during exposure time per pixel. With increasing photon count the variance of the photon count increases (Goodman, 2000). The dependency of the variance of the detected photons σ^2^ on the number of detected photons as the expected value μ can only be determined experimentally. Therefore, 100 samples of the same scene were taken and analyzed pixelwise for the variances and expected values of the measured light intensities. The resulting relationship between variance and expected value is given by σ^2^ = 3μ (cf. Supplementary Material for details). The multiplicative factor which relates variance and expected value is called *gain factor* *g* (σ^2^ = *gμ*), in our case *g* = 3. A common way to model overdispersed count data is a negative binomial model as it allows an arbitrary expectation value and variance. In general, the occurrence of *k* under a negative binomial distribution parameterized by the expected value and variance is given by

(4) P(k|μ,σ)=(k-1+μ2σ2-μk)(σ2-μσ2)k(μσ2)μ2σ2-μ

The probability to observe the light intensity *I* given the expected light intensity μ and the gain factor *g* can then be expressed as

(5) P(I|μ,g)=(I-1+μg-1I)(g-1g)Ig-μg-1

#### 2.1.3. The tilting coordinate system

We will use the coordinate system introduced by Kleiner et al. (2012): it describes the tilting stage by the direction in which the stage is tilted ψ and the actual tilting angle τ as sketched in Figure 1. For the four tilting directions carried out in the standard measurement, the tilting directions then are ψ = 0, 90, 180, 270° with a tilting angle of τ = 8°. With *N*_*Tilt*_ as the number of tilting positions the tilting directions equidistantly spanning the space are in general given by $\psi_{j}=\frac{2\pi }{N_{Tilt}}\left(j-1\right),\;j\in \left[1,2,\ldots ,N_{Tilt}\right]$ with the index *j* indicating the tilting position. The tilted vector **r_t_** is obtained by applying the appropriate rotation to the vector in the planar position **r**: **r_t_** = **R**(ψ, τ)**r**. The full rotation matrix **R**(ψ, τ) is derived by first rotating around the z-axis by −ψ, then rotating around the y-axis by τ and then rotating back around the z-axis by ψ (Wiese, 2017):

(6) $$\begin{array}{l} \text{R}(\psi ,\tau )={\text{R}}^{z}(\psi ){\text{R}}^{y}(\tau ){\text{R}}^{z}(-\psi )\\ \;=\left(\begin{array}{ccc} \cos(\psi ) & -\sin(\psi ) & 0\\ \sin(\psi ) & \cos(\psi ) & 0\\ 0 & 0 & 1 \end{array}\right)\cdot \left(\begin{array}{ccc} \cos(\tau ) & 0 & \sin(\tau )\\ 0 & 1 & 0\\ -\sin(\tau ) & 0 & \cos(\tau ) \end{array}\right)\cdot \left(\begin{array}{ccc} \cos(\psi ) & \sin(\psi ) & 0\\ -\sin(\psi ) & \cos(\psi ) & 0\\ 0 & 0 & 1 \end{array}\right)\\ \;=\left(\begin{array}{ccc} \cos(\tau )\cos{\left(\psi \right)}^{2}+\sin{\left(\psi \right)}^{2} & \left(\cos(\tau )-1)\sin(\psi )\cos(\psi \right) & \cos(\psi )\sin(\tau )\\ \left(\cos(\tau )-1)\sin(\psi )\cos(\psi \right) & \cos(\tau )\sin{\left(\psi \right)}^{2}+\cos{\left(\psi \right)}^{2} & \sin(\psi )\sin(\tau )\\ -\cos(\psi )\sin(\tau ) & -\sin(\psi )\sin(\tau ) & \cos(\tau ) \end{array}\right). \end{array}$$

As stated in Wiese et al. (2014), due to refraction at the brain tissue, the actual tilting angle in the sample is reduced and has to be adjusted according to Snell's law. Assuming a refractive index of *n* ≈ 1.45 for human tissue based on studies of de Campos Vidal et al. (1980), the tilting angle of the light in the tissue is given by $\tau_{int}\approx 5.51^{{}^{\circ}}$ for a tilt of the tilting stage by τ = 8°.

#### 2.1.4. Least-squares algorithm

We introduce the indices *j* for the tilting direction (including 0 for the planar measurement) and *i* for the rotation angle of the polarization filters. Additionaly, we denote the total number of measured tilting stage positions (tilted measurements and the planar measurement) as *N*_*T*_ = *N*_*Tilt*_ + 1 and the number of polarizer positions acquired as *N*_*P*_. All measurement data accumulates to *N*_*T*_ · *N*_*P*_ light intensities *I*_*ji*_ for each pixel. Application of the Fourier analysis to all measurements as described in Axer et al. (2011a) results in *N*_*T*_ transmittance values *I*_*j, T*_, *N*_*T*_ retardation values |sin δ_*j*_| and *N*_*T*_ direction values φ_*j*_. In this notation the intensity curve of a measurement can than be expressed as

(7) $$\begin{array}{l} I_{ji}\left(\rho_{i},\varphi_{j},\alpha_{j},d_{j}\right)=\frac{I_{j,T}}{2}\left(1+\sin\left(2\left(\rho_{i}-\varphi_{j}\right)\right)\sin\left(\frac{\pi }{2}d_{j}\cos^{2}\left(\alpha_{j}\right)\right)\right) \end{array}$$

In a first step, the effect of the average light intensity, the transmittance, is eliminated from the fiber orientation determination. Eliminating it from the further process is necessary as the light experiences additional absorption and refraction effects in a tilted measurement, which cannot be included in the model. Therefore, we define the normalized light intensity

(8) $$\begin{array}{l} I_{N_{ji}}=\frac{2I_{ji}}{I_{j,T}}-1 \end{array}$$

which shifts the data into the range [−1, 1]. As the variance of the light intensities is known as $\sigma_{I_{ji}}^{2}=gI_{ji}$ the variance of the normalized light intensity can be derived using error propagation as

(9) $$\begin{array}{l} \sigma_{N_{ji}}^{2}=\frac{gI_{ji}}{I_{j,T}^{2}}+\frac{gI_{ji}^{2}}{N_{P}I_{j,T}^{3}} \end{array}$$

The normalized light intensities *f*_*ji*_ predicted by the 3D-PLI model are given by inserting Equation (7) into Equation (8):

(10) $$\begin{array}{l} f_{ji}\left(\varphi_{j},\alpha_{j},d_{j},\rho_{i}\right)=\sin\left(2\cdot \left(\rho_{i}-\varphi_{j}\right)\right)\sin\left(\frac{\pi }{2}\cdot d_{j}\cdot \cos{\left(\alpha_{j}\right)}^{2}\right) \end{array}$$

Fitting *f* to the normalized light intensities *I*_*N*_ can now be formulated as a weighted least squares problem. With the weights $w_{ij}=\sigma_{N_{ji}}^{-1}$ the optimization problem is given by

(11) $$\begin{array}{l} \underset{\varphi ,\alpha ,d}{\mathrm{argmin}}\;\chi^{2}=\underset{\varphi ,\alpha ,d}{\mathrm{argmin}}\;\overset{N_{T}}{\underset{j=0}{\sum }}\overset{N_{P}}{\underset{i=0}{\sum }}{\left(\left(f_{ji}\left(\varphi_{j},\alpha_{j},d_{j},\rho_{i}\right)-I_{N_{ji}}\right)\cdot w_{ji}\right)}^{2} \end{array}$$

subject to $\varphi \in \left[0,\pi \right],\alpha \in \left[-\frac{\pi }{2},\frac{\pi }{2}\right],d\geq 0$. At this point we do not restrict the relative thickness to *d* ≤ 1 as a value of *d* > 1 could also occur if the current 3D-PLI model simply does not describe the data well enough. Two issues need to be overcome to solve the optimization problem: the bounded parameter space and a suitable starting point for the local optimization. These issues resemble the optimization problem of the maximum likelihood algorithm described in Wiese (2017).

A starting point for the direction angle is given by the direction angle derived from the planar measurement φ_0_. For the inclination α and the relative thickness *d*, the starting point is determined by brute force minimization. Based on simulation studies, a 6 × 6 grid equidistantly spanning the parameter space ([φ_0_, α_*l*_, *d*_*k*_], *k, l* = 1, …, 6) was found to result in a promising first guess for the subsequent optimization.

The boundaries of the parameter space could be accounted for by utilizing an optimization algorithm capable of dealing with hard boundaries. Yet, in our case a more elegant solution is to exploit the symmetry of the problem. This enables to reformulate the bounded optimization problem as an unbounded problem. As *f*(φ, α, *d*) = −*f*(φ, α, −*d*), it is sufficient to allow all relative thicknesses *d* and take the absolute value of the relative section thickness *d* = |*d*| before calculating the normalized intensities. For the fiber orientations, it is also possible to allow all orientations considering the symmetry of spherical coordinates. The unbounded orientation given as (φ_*u*_, α_*u*_) can be transformed back into the standard 3D-PLI parameter space by the following transformations:

(12) $$\begin{array}{l} \alpha =\left(\left(\alpha_{u}+\frac{\pi }{2}\right)\text{ mod }\pi -\frac{\pi }{2}\right)\text{ sgn}\left(\frac{1}{2}-\left\lfloor \frac{\varphi_{u}}{\pi }\text{mod }2\right\rfloor \right) \end{array}$$

(13) $$\begin{array}{l} \varphi =\varphi_{u}\text{ mod }\pi \end{array}$$

Before calculating the normalized light intensities *f*, these transformations are used to symmetrize the unbounded orientation into the parameter space. As optimization algorithm the Levenberg-Marquardt algorithm was chosen (Levenberg, 1944; Marquardt, 1963). In case of convergence to a point outside of the parameter space, the before mentioned transformations are used to symmetrize the result back into the parameter space. This newly developped algorithm is denoted as *Robust Orientation Fitting via Least Squares (ROFL)*. A pseudocode is given in Algorithm 1.

**Algorithm 1:**
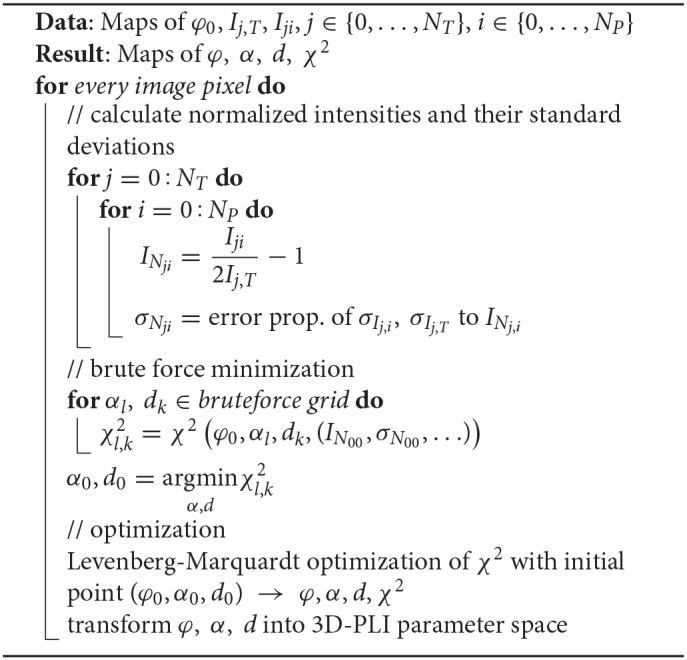
Pseudocode of the ROFL algorithm

For this study, the ROFL algorithm was implemented in *Python* based on the packages *NumPy* (van der Walt et al., 2011) and *SciPy* (Jones et al., 2001). The necessary computation time is very high as the optimization has to be carried out in every image pixel (about 4 core hours for an image size of ≈ 10^6^ pixels). Therefore, the algorithm was parallelized pixelwise using *mpi4py* (Dalcin et al., 2005) and all calculations were conducted on the JURECA system (Jülich Supercomputing Centre, 2016).

### 2.2. Simulated data

Monte-Carlo simulations were carried out to test the robustness of the presented approach and the DFT algorithm against measurement noise.

#### 2.2.1. Generation of synthetic data

Simulations can provide information about two problems. Firstly, the accuracy of the obtained parameters can be compared to the synthetic ground truth. Secondly, the algorithms can be tested against biases in their parameter estimation. One sythetic dataset each was created to answer these questions. For both datasets, the generation of synthetic 3D-PLI signals for a specific parameter set is executed in the same way.

Synthetic signals were generated in a similar manner as in Wiese et al. (2014). A ground truth orientation vector **v**_*g*_ with the parameters (*d*_*g*_, φ_*g*_, α_*g*_) was rotated into four tilting positions (North, South, East, West) by τ = 5.51°, the assumed internal tilting angle for human brain tissue. For each tilting position a sinusoidal light intensity profile according to Equation (1) was calculated. For the transmittance *I*_*T*_ 2500 was chosen, as it represents a typical transmittance value for human brain sections. To mimic the experimentally observed light intensity distribution, for each calculated light intensity *I* a noisy light intensity *I*_noisy_ was then computed by drawing one sample from a negative binomial distribution with expected value given by *I* and variance 3*I*: $I_{\text{noisy}}\propto \mathrm{NB}\left(I,3I\right)$.

In a first simulation, the accuracy of the obtained parameters was assessed. As our primary interest here was the validation of the reconstruction of the inclination and the relative section thickness, only one direction angle, φ = 45°, was simulated. This direction angle also represents the worst case scenario for the four tilting positions as the angle between the orientation vector and the rotated orientation vector is maximal for φ = ψ and decreases with |φ − ψ|. With respect to the inclination angle, it is sufficient to simulate only positive inclinations as the inclination sign does not effect the reconstruction precision. As ground truth vectors all combinations of the fixed direction angle 45°, inclinations α from 0°, 1°, …, 89°, 90°, and thicknesses *d* from 0.01, 0.02, …, 0.89, 0.9 were simulated. These fiber configurations display all different inclination angles for different tissue scenarios characterized by the relative thickness *d*. For each ground truth vector 100,000 samples of 3D-PLI signals were generated utilizing the beforementioned method to enable a statistical analysis.

To tests the algorithms against biases in their inclination estimation, a second dataset of 500,000 samples of orientation vectors uniformly distributed on the unit sphere were computed. From these vectors, a dataset of direction and inclination angles was derived and used to calculate the noisy sinusoidal light intensity profiles with a relative thickness of *d* = 0.5.

#### 2.2.2. Evaluation

The simulated sinusoidal profiles were analyzed with the ROFL algorithm and the DFT algorithm resulting in the reconstructed vector **v**_*r*_ with the parameters (*d*_*r*_, φ_*r*_, α_*r*_). The accuracy of the obtained orientation was then measured by the acute angle γ between the ground truth vector **v**_*g*_ and the reconstructed vector **v**_*r*_:

(14) $$\begin{array}{l} \gamma =\arccos\left(\left|{\text{v}}_{r}\cdot {\text{v}}_{g}\right|\right) \end{array}$$

The acute angle respects the symmetry of the problem: as the parameter space is bounded to a half sphere, the maximal angle between two vectors is 90°. Therefore, the absolute value of the scalar product is used in Equation (14). For each ground truth vector, the overall reconstruction error is given by the mean deviation angle 〈γ〉 of all 100,000 samples. The reconstruction accuracy of the relative thickness *d* is evaluated by the absolute relative error between the obtained values and the ground truth: $\sigma_{d}=|\frac{d_{r}-d_{g}}{d_{g}}|$. The overall error for a fiber configuration is then again given by the mean relative error of all 100,000 samples 〈σ_*d*_〉.

To assess if the obtained inclinations are biased, inclination angles were reconstructed from the second synthetic dataset. The resulting inclination angle histograms were then compared to the ground truth inclination histogram.

### 2.3. Experimental data

To test and compare the DFT and the ROFL algorithm on experimental data, a series of coronal sections of a right human hemisphere was investigated. The post mortem human tissue sample used for this study was acquired in accordance with the local ethic committee of our partner university at the Heinrich Heine University Düsseldorf. As confirmed by the ethic committee, postmortem human brain studies do not need any additional approval, if a written informed consent of the subject is available. For the research carried out here, this consent is available.

#### 2.3.1. Data acquisition

The examined brain was removed within 24 h after the donor's death. The right hemisphere was fixed in 4% buffered formaldehyde solution for 15 days to prevent tissue degeneration. After immersion in a 20% solution of glycerin with Dimethyl sulfoxide (DMSO) for cryoprotection the brain was frozen at a temperature of −80°C. The coronal cutting plane was decided to be orthogonal to a line connecting the anterior and posterior commissures. Before sectioning, the frontal lobe was cut off to create a stable cutting platform. The sectioning resulted in 843 sections of 70 μm thickness (*Polycut CM 3500, Leica, Germany*). Before cutting an image of the cutting surface (*blockface image*) was taken for each section. Each section was then mounted on a glass slide, immersed in a 20% solution of glycerin to avoid dehydration and sealed by a coverslip. All sections were measured with the mentioned setup with the standard measurement protocol of the planar measurement and four tilting positions.

#### 2.3.2. Data analysis

After an intensity based calibration as described in Dammers et al. (2010) the Fourier analysis was utilized to obtain the standard 3D-PLI modalities transmittance, retardation and direction. Then the ROFL algorithm was used to extract maps of the direction and inclination angles, the relative section thickness *d* and the residuum χ^2^. As a comparison, the DFT algorithm was applied to determine the inclination angle and relative section thickness. Also, the residuum was calculated for the parameters obtained by DFT.

After the sectionwise analysis, the 3D-reconstruction of the measured sections requires a multi-step registration to retain the 3D volume of the measured brain. Here the blockface volume generated by aligning the blockface images to a 3D volume serves as a reference for the registration of the histological sections (Schober et al., 2015). In a first step, the histological sections are registered onto their corresponding blockface images via an affine transformation utilizing in-house developed software based on the software packages *ITK* (Yoo et al., 2002) and *elastix* (Klein et al., 2009). Then non-linear registration using the ANTs toolkit (Avants et al., 2011) was performed. Finally, the scalar modalities were spatially transformed using the obtained deformation fields. The direction angles were rotated according to the pixelwise rotations induced by the deformation computed by the nonlinear image registration. Pixel values were linearly interpolated. The reorientation can result in orientations lying outside of the parameter space, therefore the same mapping as in ROFL (see Equation 13) was applied after the reorientation.

#### 2.3.3. Validation

Traditional anatomical studies have described the fiber pathways globally and without considering differences between brains. At the level of detail presented in this study with a voxel size of 64 × 64 × 70μm^3^, the inter-subject variability in the fine structure of fiber orientations becomes much more relevant. Therefore, the resulting fiber orientations cannot easily be compared to anatomical atlases. A complementary measurement at the same resolution enabling a comparison is not available either. Additionally, a phantom for 3D-PLI has not been developed yet, impeding the possibility of a measurement with a known ground truth. Therefore, we chose to validate the resulting orientations based on their coherence across the whole volume and their alignment to anatomical boundaries, for example, within the sagittal stratum. The results of both algorithms were furthermore compared from a statistical point of view by the difference between the predicted and measured light intensities measured by the sum of the squared residuals.

## 3. Results

### 3.1. Simulation

#### 3.1.1. Accuracy evaluation

**Figure 2:**
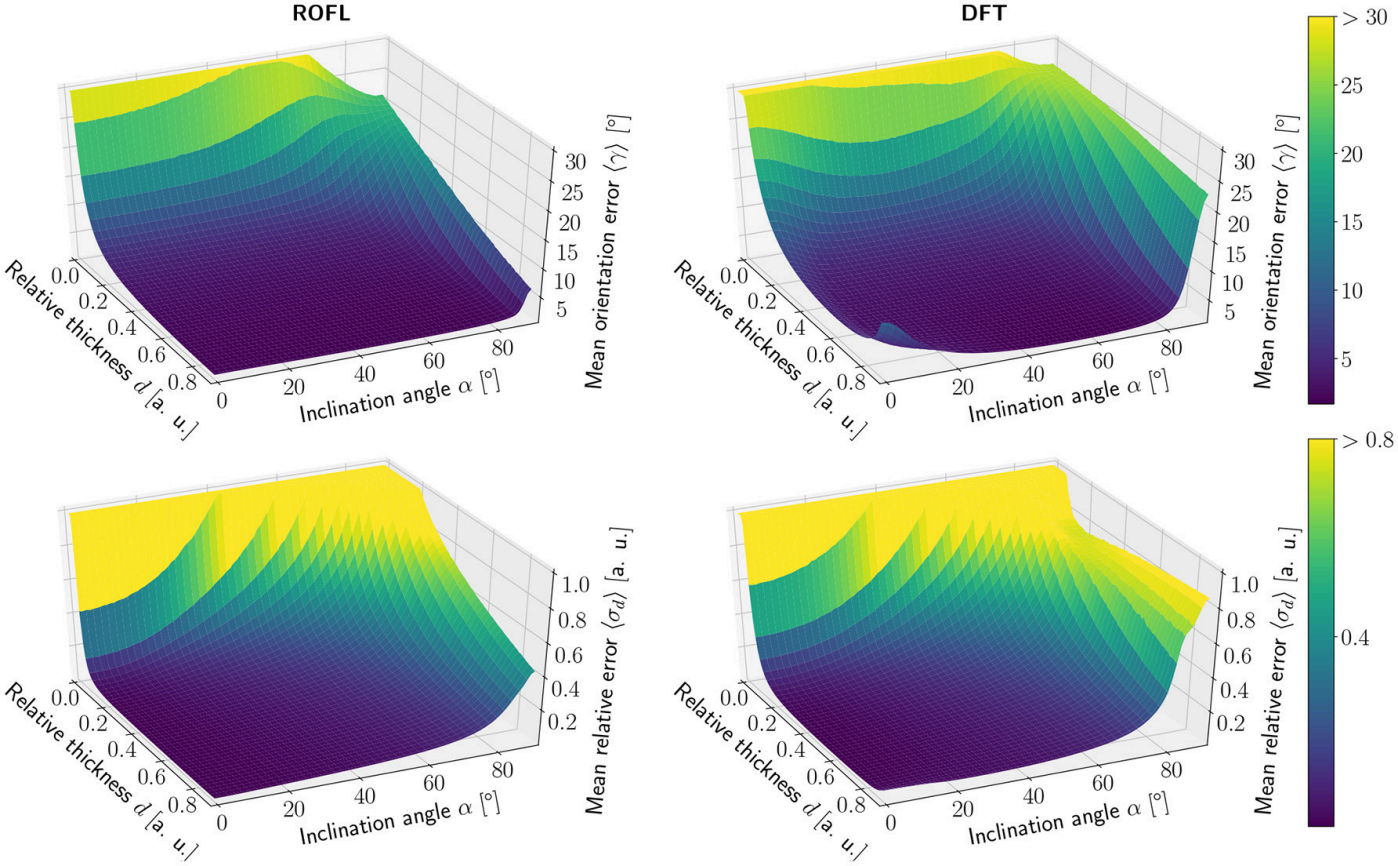
Simulation results. **(Left)** ROFL algorithm, **(Right)** DFT algorithm. **(Top)** Orientation reconstruction error 〈γ〉 as a function of relative thickness *d* and inclination angle α. For illustration purposes the z-axis is clipped at 30°. **(Bottom)** relative reconstruction error of the relative thickness 〈σ_*d*_〉 as a function of relative thickness *d* and inclination angle α. For illustration purposes the z-axis is limited to [0, 1].

The simulation results of the first simulated dataset are depicted in Figure 2: The plots show the orientation reconstruction error and the relative reconstruction error of the relative thickness of both algorithms as a function of inclination angle α and relative thickness *d*.

##### 3.1.1.1. Orientation reconstruction

The orientation reconstruction accuracy of the DFT is valley shaped with respect to the inclination angle and becomes minimal for an inclination angle of α ≈ 60°. From this minimum the orientation reconstruction error increases slightly for flat fibers. The most challenging orientations to analyze are very steep fibers with respect to the sectioning plane: for these, the orientations predicted by the DFT algorithm differ strongly from the ground truth. Even for relative thicknesses of *d* > 0.2 an average reconstruction error of 〈γ〉 ≈ 22° occurs for α > 80°. With decreasing relative thickness *d* the reconstruction accuracy also decreases: for *d* < 0.05, the obtained orientations are basically random.

In contrast, the orientation reconstruction error for the ROFL algorithm does not take the form of a valley: for all section thicknesses *d* > 0.06, the error is minimal for in-plane fibers with α = 0° and increases with the inclination angle. For very steep fibers of α > 80°, the error increases to γ ≈ 12° on average. As before, the reconstruction error increases with decreasing relative thickness *d*. In a direct comparison, the orientation reconstruction error achieved by the ROFL algorithm is lower than the orientation reconstruction error achieved by the DFT algorithm for all configurations. The minimal error for the ROFL algorithm is given by 〈γ〉 = 1°, compared to 〈γ〉 = 1.6° for the DFT algorithm. For *d* ∈ [0.2, 0.9] and α ∈ [0°, 80°], the orientation reconstruction accuracy achieved by the algorithms are on average 2° for ROFL and 4.5° for DFT with maximal values of 9.5° for ROFL and 18.8° for DFT.

##### 3.1.1.2. Reconstruction of the relative section thickness

Similar to the the orientation reconstruction error, the relative reconstruction error 〈σ_*d*_〉 of the relative thickness *d* increases with the inclination angle. Here, the errors obtained from the DFT algorithm do not assume the shape of a valley: besides a noise instability for very high relative thicknesses *d* > 0.8 the error increases with the inclination and increases with decreasing relative thickness *d*. The relative reconstruction error of the ROFL algorithm assumes the same shape but is lower: on average, the relative thickness of fibers with *d* ∈ [0.2, 0.9] and α ∈ [20°, 90°] is determined with an error of 5% compared to 10% for the DFT algorithm. The most challenging fiber configurations to reconstruct are again very low relative thicknesses and very steep fibers: for *d* < 0.1 and α < 16°, the relative error amounts to 〈σ_*d*_〉 > 1 for both algorithms. For very steep fibers, the error observed in the results of the DFT algorithm increases to up to 80%. The ROFL algorithm achieves a lower error in this case, yet the best case for *d* = 0.9 still expresses a relative error of 〈σ〉 ≈ 42%.

#### 3.1.2. Inclination bias evaluation

As the orientation vectors were distributed uniformly on a sphere, the frequency of the ground truth inclinations is proportional to the circumference of the cross section of the sphere at the respective height corresponding to a given inclination. In our definition of the inclination angle as $\alpha \in \left[-\frac{\pi }{2},\frac{\pi }{2}\right]$, the circumference of the cross section of the unit sphere at a respective inclination is proportional to cos(α). In consequence, the inclination frequency *p*(α) is expected to be proportional to cos(α): *p*(α) ∝ cos(α).

**Figure 3:**
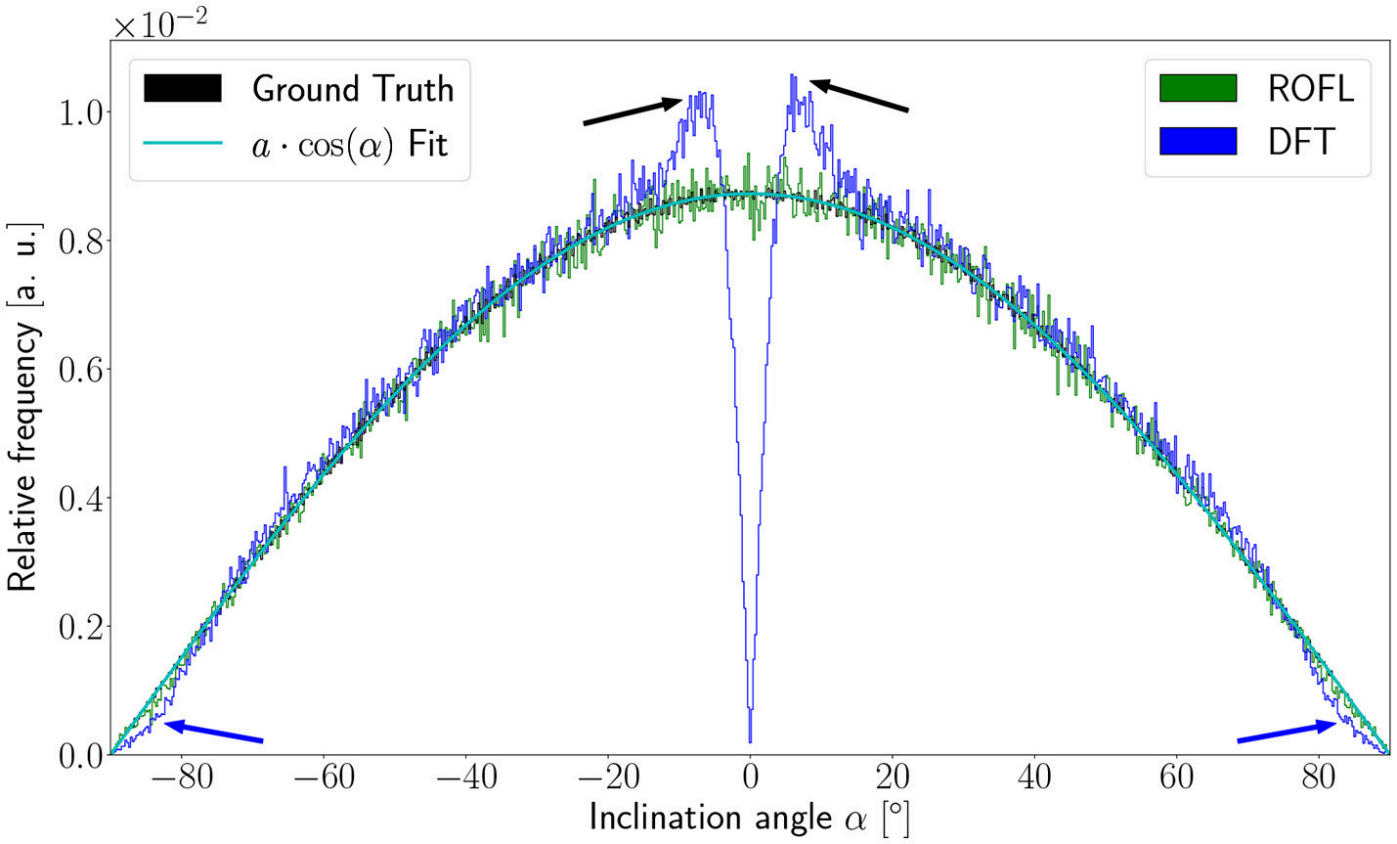
Inclination histograms for uniformly distributed orientations obtained from ROFL and DFT algorithms and ground truth. The magenta line depicts a fit curve to the ground truth inclination histogram. Fit result: *a* = 0.0087. The black arrows highlight peaks for in-plane orientations, the blue arrows point out differences between the DFT histogram and the ground truth for out-of-plane orientations. Bin width: 0.25°.

The inclination histograms obtained from the simulated dataset of uniformly distributed orientation vectors are depicted in Figure 3. The histogram of the ground truth inclinations does indeed follow the expected cos(α) proportionality as can be seen from the *a*·cos(α) curve fit with proportionality factor *a* in cyan. The proposed ROFL approach achieves a high agreement with the ground truth inclination. On the other hand, the histogram of the inclinations computed by the DFT algorithm reveals a severe lack of in-plane inclinations. Especially orientations with α = 0° which are the most probable orientations are barely computed at all. Instead of an increasing density toward 0°, the histogram displays two symmetric peaks left and right of 0° indicated by black arrows which are not present in the ground truth. Another observation lies in the frequency of very steep fibers with respect to the sectioning plane: for |α| > 80°, the frequency of DFT inclinations decreases moderately as indicated by blue arrows.

### 3.2. Experimental data

**Figure 4:**
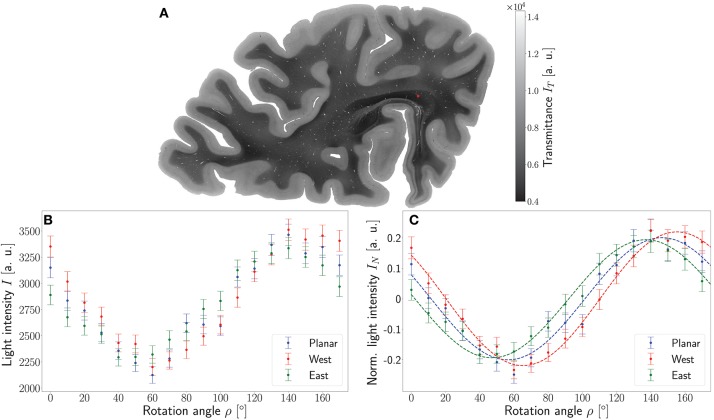
Working principle of the ROFL algorithm demonstrated for a single pixel. **(A)** Transmittance map. The red circle points out the position of the analyzed pixel. **(B)** Measured light intensities of the planar measurement and tilted measurements to west and east after calibration and registration onto the planar measurement. **(C)** Normalized light intensities of the planar measurement and tilted measurements to west and east. The dashed lines depict their best fit curves according to the ROFL algorithm. Fit result: φ = 101°, α = −60°, *d* = 0.5, *R*^2^ = 0.97.

To demonstrate the working principle of the ROFL algorithm by means of experimental data, one pixel of the stratum sagittale highlighted in Figure 4A was chosen. The obtained measurement data from the planar and two tilted measurements after calibration and coregistration are plotted in Figure 4B. Figure 4C then shows the normalized light intensities and their best fit curves according to the polarimetric model as predicted by ROFL.

**Figure 5:**
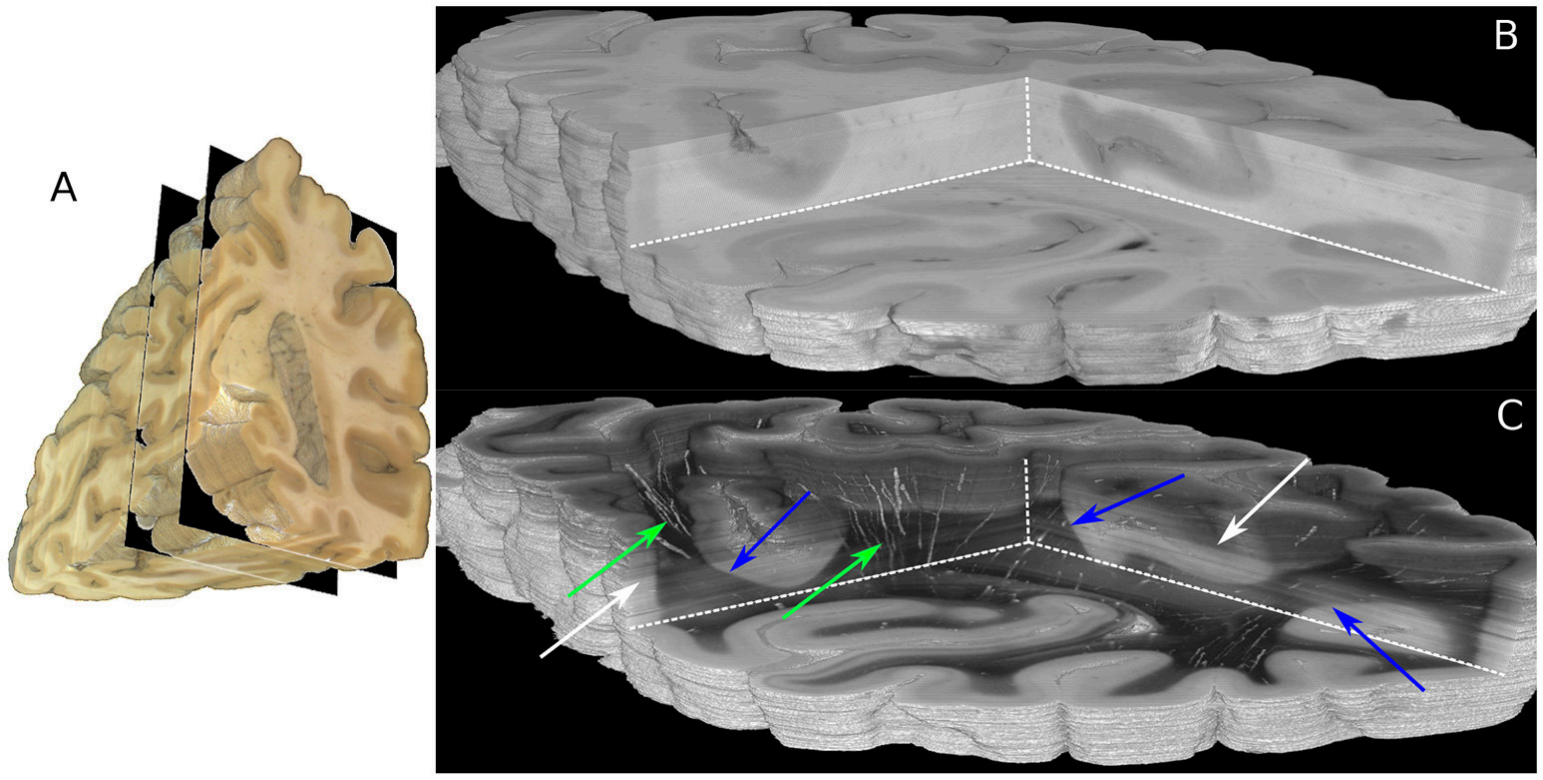
Reconstructed 3D-PLI volumes. **(A)** Full blockface volume with black planes representing the boundaries of the analyzed sections shown in **(B,C)**. **(B)** Partial blockface volume of the histologically analyzed and reconstructed sections. **(C)** Transmittance volume reconstructed from the histological sections. The green arrows highlight reconstructed blood vessel structures. Note, for the reasons of clarity only vessels with very strong birefringence signals are shown here. The blue arrows indicate white/gray matter transition zones. Note that brightnesses variations pointed out by white arrows occur due to differing times between mounting of the section on the glass slide and the 3D-PLI measurement.

The boundaries of the investigated 230 coronal sections are illustrated in the view of the blockface volume shown in Figure 5A (Schober et al., 2015; Wiese, 2017). A comparison of the reconstructed histological sections and the corresponding part of the blockface volume is depicted in Figures 5B,C utilizing the clipping box view technique implemented in the *PLIVIS* tool (Schubert et al., 2016, 2018). As the blockface images capture the reflected light during brain sectioning and the transmittance image the transmitted light, they appear inverted to each other. As the time between section mounting and measurement was not constant for all sections, the transmittance differs over the volume as indicated by the white arrows in Figure 5C. The reconstruction precision is demonstrated by the smooth reconstruction at the white matter/gray matter transition zones, but also by the fine-grained blood vessel structures highlighted in green in Figure 5C.

**Figure 6:**
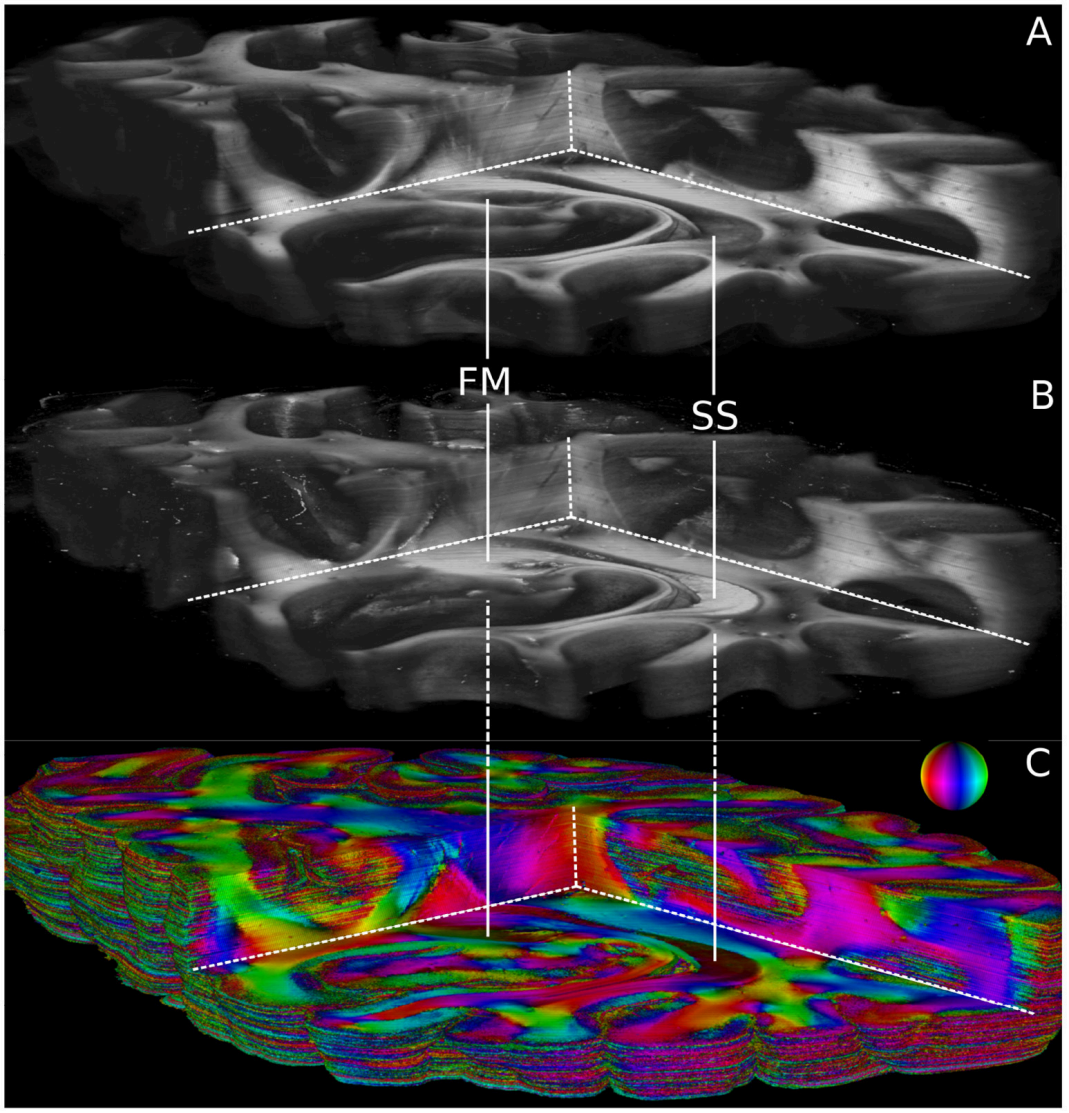
Reconstructed 3D-PLI modalities. **(A)** Retardation volume, **(B)** Relative section thickness volume computed by the ROFL algorithm and denoised section-wise with a median filter of radius 1. **(C)** Fiber orientation volume computed by the ROFL algorithm. The vector-valued fiber orientations are encoded in colors and indicated by the color sphere. FM, forceps major; SS, stratum sagittale.

Volumetric views of 3D-PLI modalities are shown in Figure 6. For clarity, the view is the same as in Figure 5. The modalities retardation |sinδ| (cf. Figure 6A) and relative section thickness *d* (cf. Figure 6B) revealed a strong agreement in most brain regions of the reconstructed volume. Differences, however, were observed in particular for white matter fiber tracts characterized by low retardation values. Those tracts took courses (close to) perpendicular to the sectioning plane according to the fiber orientation map shown in Figure 6C. The forceps major (FM) and the stratum sagittale (SS) were worked out as prominent examples for such tracts (cf. Figure 6). Ultimately, the obtained fiber orientations (Figure 6), in particular in deep white matter structures appeared coherent across neighboring sections. Fiber tracts and pathways were clearly distinguishable from each other and could be traced through the reconstructed volume.

#### 3.2.1. Comparison of ROFL and DFT algorithms

**Figure 7:**
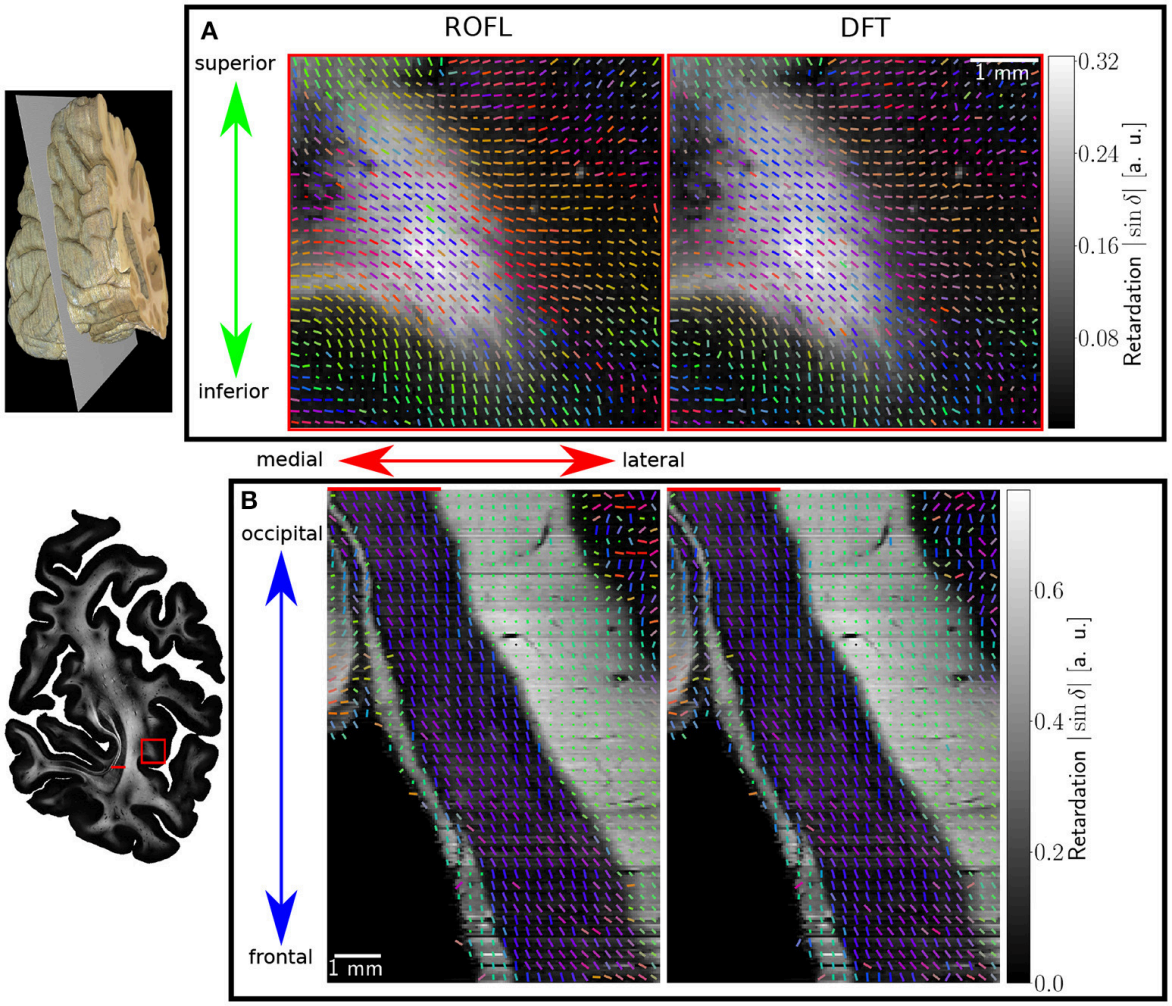
Vectorfields obtained with the ROFL algorithm (left) and the DFT algorithm (right) underlayed with the retardation map. **(A)** Region of interest at the boundary of white and gray matter, indicated by the red rectangle in the retardation map. Every third vector is mapped. **(B)** Region of interest in the stratum sagittale, resliced view of the volume at the position indicated by the red line. Every fifth vector is mapped.

**Figure 8:**
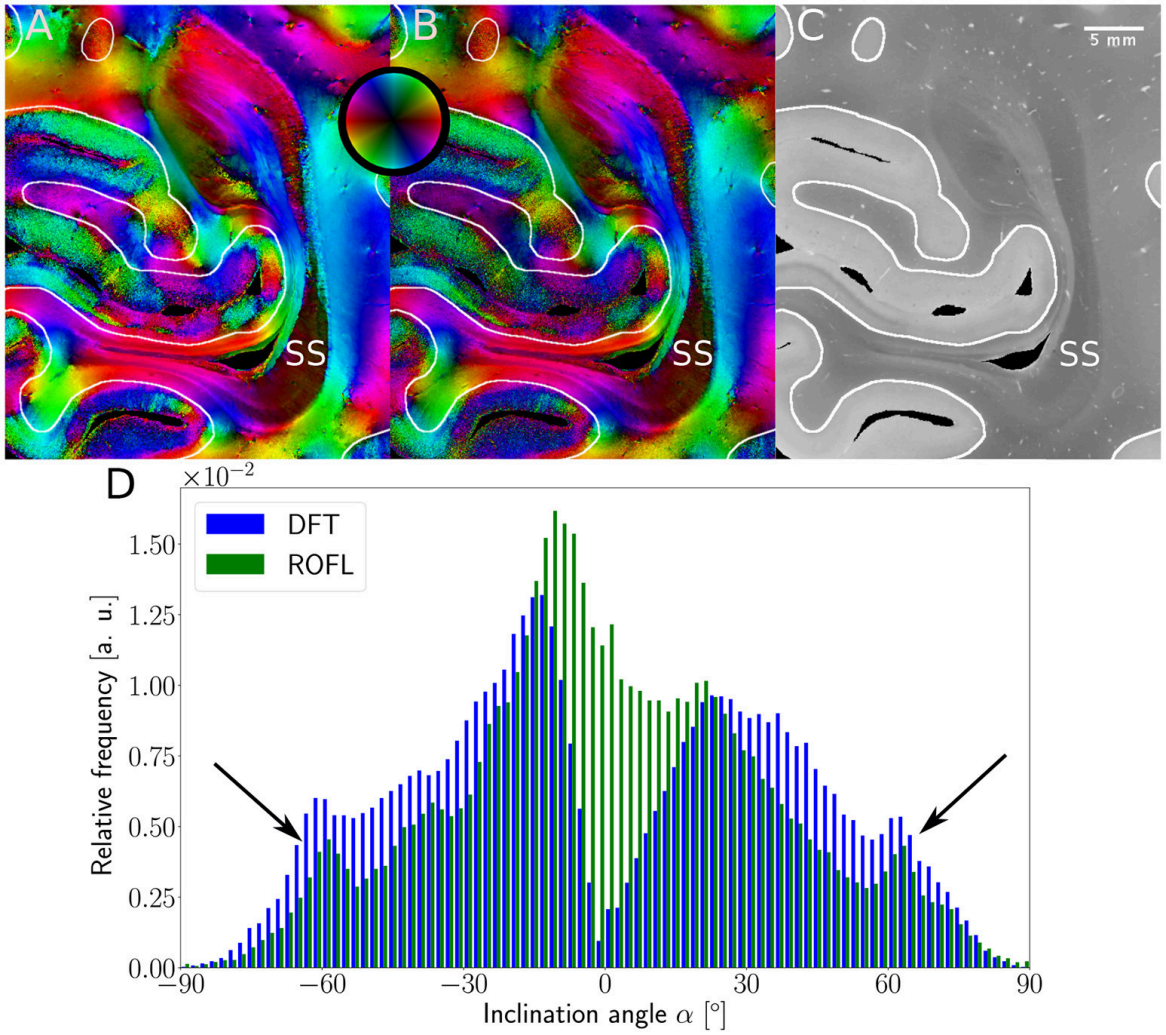
3D-PLI modalities of a representative brain section. **(A)** Fiber orientation map in the region of the stratum sagittale derived with the ROFL algorithm. **(B)** Fiber orientation map resulting from the DFT algorithm. **(C)** Transmittance map. The white lines represent a manual delineation of the white/gray matter transition zones. SS, stratum sagittale. **(D)** Histogram of the inclination angles of white matter pixels obtained from DFT and ROFL algorithms. The arrow points out peaks of the histogram for steep fibers with respect to the sectioning plane. Bin width: 2°.

Major differences between the ROFL algorithm and the DFT algorithm were particularly observed for two cases: low relative section thickness and very steep fibers with respect to the sectioning plane. Therefore, the vector fields of two regions were investigated more closely: the stratum sagittale which is expected to run perpendicular to the sectioning plane and a region at the boundary of white and gray matter. The vector fields underlaid with the retardation maps are shown in Figure 7. The plotted two-dimensional, colored lines are representations of the projections of three-dimensional fiber orientation vectors into the respective plane, color-coded by the 3D orientation. The region of interest at the transition zones of white and gray matter shown in Figure 7A. Both vector fields are very similar, but the ROFL algorithm results in less inclined orientations than the DFT approach. As the plotted vectors represent the projection of the three-dimensional fiber orientation vectors into the respective plane, longer vectors imply flatter fibers with respect to the respective plane which is the case here. The vector field in Figure 7B shows the xz plane perpendicular to the coronal sectioning plane to highlight the robust reconstruction of the stratum sagittale. Minor differences are visible at the boundary of the stratum sagittale but overall both algorithms agreed well. As on simulated data the inclination histograms exposed a distinct lacking of in-plane fiber orientations with respect to the sectioning plane for the DFT algorithm, the inclination histograms obtained from experimental data were also investigated. For this purpose one region of interest from the white matter of one section was examined. The fiber orientation maps (FOMs) resulting from ROFL and DFT are shown in Figures 8A,B, respectively. A general glance revealed similar orientation values for both algorithms, which holds true for white and gray matter regions. Due to the considerable amount of noise in the fiber orientations in gray matter, only white matter pixels were considered for the inclination histograms. A detailed analysis of the inclination angle histograms of the white matter regions delineated in Figure 8C yielded the following observations (cf. Figure 8D): while the frequency of inclination angles drastically decreases from −15° to 0° and increases again to 20° for the DFT algorithm, this lacking of orientations was not observed for the ROFL algorithm. For the regime of high absolute inclination angles, both histograms agree: both have peaks for inclination angles of ≈ ± 60° (cf. arrows in Figure 8D) which result from fibers oriented perpendicular to the sectioning plane in the stratum sagittale (cf. Figures 8A–C).

In addition to the potentially biased visual inspection of the vector fields, an unbiased statistical statement about the reconstruction accuracy is given by the mean residual: the mean value of the mean residuals of all pixels is 15% lower for the results of the ROFL algorithm than the results of the DFT algorithm.

#### 3.2.2. Agreement of model and data

**Figure 9:**
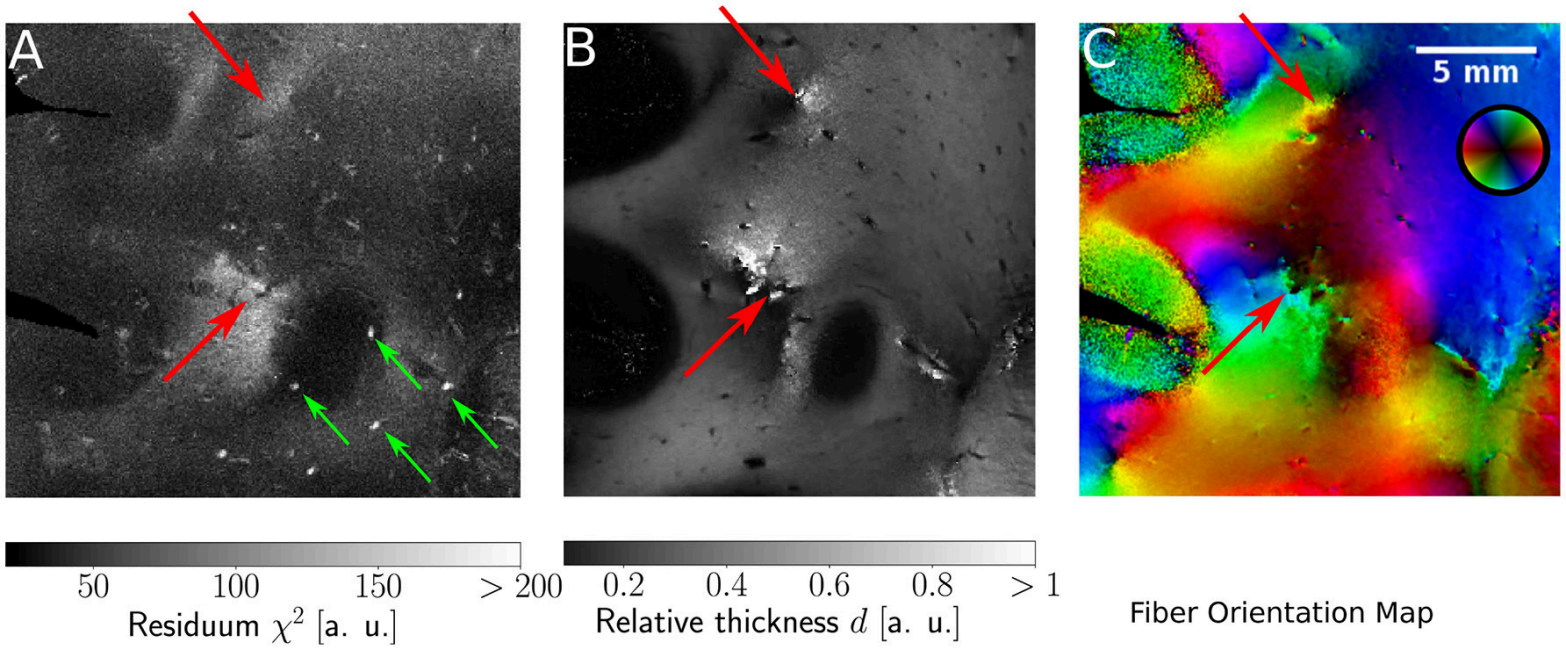
Identification of artifacts and fiber crossings. **(A)** Residuum map χ^2^ with green arrows indicating dust particles. **(B)** Relative thickness map *d*. **(C)** Fiber Orientation map. Red arrows indicate fiber crossings.

Since the ROFL algorithm performs a least squares fit in all image pixel, the actual sum of residuals χ^2^ (as a result of the optimization process) was accessible. For the DFT algorithm the residual map can also be calculated, yet this requires additional computations which take even longer than the runtime of the algorithm itself. Figure 9 shows maps of the residuum χ^2^ (Figure 9A), the relative thickness *d* (Figure 9B) and the fiber orientation map (Figure 9C) for a selected region of interest. As χ^2^ is a measure of the difference between the model and the experimental data, it is expected to increase for artifacts such as dust particles which rotate with the polarization filters. Such artifacts were indeed observed in experimental results (cf. Figure 9A, where dust particles are indicated by green arrows). For one pixel, a dust particle corrupts one of the 18 obtained values during filter rotation. Still it does not necessarily have a strong effect on the resulting fit values as the corresponding relative thicknesses and fiber orientations do not show signs of artifacts. The χ^2^ measure, however, is sensitive to such artifacts.

In addition, we found areas of increased residuals (cf. red arrows in Figure 9) we were able to assign to crossing fiber regions. In the fiber orientation map, crossings stood out as the center of an abrupt change of colors (Figure 9C). Also, the optimization process resulted in an abrupt change of the relative thickness, even with values of *d* > 1 for which the relationship between the retardation and inclination is not bijective anymore (Figure 9B). Due to the bad agreement between the model and the data, the residuum finally increased strongly (cf. red arrows in Figure 9).

## 4. Discussion and outlook

We introduced the least-squares algorithm ROFL for the reconstruction of fiber orientation and the extraction of the relative section thickness from measurements with a tiltable specimen stage in 3D-Polarized Light Imaging (3D-PLI). This method requires only one additional assumption besides the polarimetric model: the refractive index of the brain tissue. This represents a substantial improvement as compared to other histological imaging techniques which strongly rely on parameter dependent image processing pipelines to extract three-dimensional fiber orientations. To our knowledge, no other histological imaging technique is currently capable of deriving three-dimensional information from a biophysical model. This compensates for the disadvantage of the difficulties accompanying the 3D re-alignment of serial high-resolution brain section images as required for 3D-PLI anaylsis.

The working principle of the ROFL algorithm was proven for simulated data for which it resulted in a significant improvement of the orientation reconstruction. It was opposed to a previously implemented analytical algorithm based on a discrete Fourier transformation (DFT). The reconstruction with ROFL became more reliable, in particular for low relative section thicknesses. The noise instability of the DFT algorithm for very flat fibers was already observed by Wiese et al. (2014) and is explainable by the fact that for very flat fibers the gradient $\frac{\partial \sin\delta }{\partial \alpha }$ becomes small (for in-plane fibers with α = 0° it even becomes zero: $\frac{\partial \sin\delta }{\partial \alpha }{|}_{\alpha =0^{{}^{\circ}}}=0$). This makes the DFT algorithm prone to noise effects in this case. In fact, the simulations proved that the DFT algorithm is not capable of reconstructing in-plane orientations. On the contrary, the presented fitting algorithm still enabled a reliable reconstruction for in-plane fibers. The random orientations computed by the DFT algorithm for small relative section thicknesses of *d* < 0.05 were not surprising, as for *d* < 0.05, the maximal possible retardation value is also |sin δ| = 0.05 and the retardation values of the different tilting positions become almost indistinguishable. In this case, the ROFL algorithm provides a superior reconstruction. In the case of very steep fibers, the ROFL algorithm also outperformed DFT significantly. Still, the reconstruction accuracy decreases strongly for very steep fibers also for the ROFL algorithm. This originates from the signal itself: for α = 90°, the amplitude of the sinusoidal signals are almost zero and the measured signals resemble a constant function with random noise (cf. Dohmen et al., 2015 for a theoretical description of this issue). While the reconstruction of out-of-plane fibers remains challenging, orientation vectors with |α| > 80° only make up approx. 3% of all possible orientations. Therefore, we can conclude that ROFL accomplishes a very robust orientation reconstruction with an average accuracy of 2° for the vast majority of possible fiber orientations for relative section thicknesses of *d* > 0.2 on synthetic data.

ROFL was applied to 230 consecutive human brain sections subjected to 3D-PLI. The results were 3D reconstructed to demonstrate the robustness and reproducibility of our approach. The obtained modality volumes of retardation, relative section thickness and fiber orientation were characterized by high coherence across the sections. Anatomical structures, such as fiber tracts, white/gray matter borders, vessels could be reconstructed with high precision. The transmittance volume displayed brightness variations, yet the strength of our approach is that the determination of the fiber orientation and relative section thickness is independent of the transmittance due to the normalization. By eyesight, no major differences were observed in the vector fields resulting from the ROFL and DFT approaches. In the stratum sagittale, both approaches resulted in very similar orientations with very minor differences. The computed inclinations were on average α ≈ 65° and the computed relative section thicknesses *d* = 0.6. This combination actually corresponds to the minimum of the orientation reconstruction error of the DFT algorithm obtained from the simulated datasets, which suggests that a strong agreement with the ROFL algorithm can be expected. At the boundary of white and gray matter regions, the ROFL algorithm resulted in less inclined fiber orientations than DFT but also only very minor differences were observable.

The inclination histograms, however, yielded a very important finding: whereas, the frequency of the computed inclination angles decreased significantly for in-plane fibers with α ≈ 0° for the DFT algorithm, the ROFL algorithm was still capable of reconstructing the whole spectrum of possible inclinations. The same behavior was observed for simulated data, which proves a systematic bias of the DFT algorithm. The ROFL result is also more plausible as for the observed ROI in Figure 8 the inclination histogram obtained from ROFL agrees far better with the inclination histogram of uniformly distributed orientations. Still, it has to be noted that fiber orientations in a small ROI of the brain cannot expected to be perfectly uniformly distributed due to the convoluted structure of the human brain. The observed inclination differences between ROFL and DFT are hard to observe based on orientation vectors, as the actual inclination differences of up to 10° for in-plane orientations can barely be distinguished by eye. As the difference between the measured and predicted light intensities according to the 3D-PLI model also decreases significantly for the parameters obtained from ROFL compared to DFT, ROFL yields more accurate results than DFT also from a purely statistical perspective.

Furthermore, the ROFL algorithm enables a direct way to evaluate the agreement of model and data based on the residuum map which would otherwise have to be computed additionally. This kind of map was exploited to identify measurement artifacts and, even more importantly, crossing fiber regions which were not classifiable by previous 3D-PLI analysis approaches (Axer et al., 2011a; Kleiner et al., 2012; Wiese et al., 2014). Fiber crossings currently pose the greatest complication for 3D-PLI analysis as partial volume effects are still present in brain tissue at voxel sizes of 64 × 64 × 70μm^3^. A voxel containing fibers with different courses (i.e., orientations) will inevitably result in a 3D-PLI measurement composed of superimposed birefringence signals. This might lead to a biased orientation interpretation. To give an example, a voxel comprising crossing out-of-plane and in-plane fibers, the ROFL algorithm will preferably reconstruct the orientation which causes a larger signal, in this case the in-plane orientation. To overcome this issue, different strategies are currently being explored: (i) Modeling of crossing fiber structures and subsequent simulations of tilting measurements utilizing the simPLI simulation platform introduced by Dohmen et al. (2015). By this means the behavior of the ROFL algorithm in case of well-known crossing fiber constellations can be further investigated. (ii) Realizing the idea of oblique imaging for in-plane resolutions at the micrometer scale. Preliminary measurements of a mouse section using a prototypic “tilting” polarizing microscope built up at an optical bench (pixel size: 1.3 × 1.3μm) already revealed the benefit of smaller voxel sizes.

While the obtained parameters show a high coherence across the volume, no quantitative statement about the reliability of the resulting parameters is possible at this point. Hence, future studies need to investigate the uncertainty of the fitted parameters. For Diffusion Tensor Imaging, for example, bootstrapping approaches were used to obtain orientation confidence maps (Jones, 2003; Heim et al., 2004; Whitcher et al., 2008). Especially, the uncertainty of the relative section thickness is of interest, as this parameter is as an indicator for myelin and might even be a reliable measure of the local myelin density. As a matter of fact, the relative section thickness is proportional to the local birefringence Δ*n* which was clearly attributed to the myelin sheath in earlier studies (Goethlin, 1913; Schmidt, 1923; Schmitt and Bear, 1937; de Campos Vidal et al., 1980). Since myelin density information is of outmost interest for the research of degenerative brain diseases characterized by locally altered myelination (e.g., multiple sclerosis), future studies will address the correlation between the relative section thickness and myelin density.

The improved reconstruction comes at the cost of computation time, which increases by a factor of 5,000. Yet, as processing a single brain section takes about 3 min using four compute nodes on JURECA (Jülich Supercomputing Centre, 2016) with the ROFL algorithm in its current implementation, even the computation of whole brains becomes feasible. Still, the computation time could further be reduced by utilizing GPU ressources (Przybylski et al., 2017).

To conclude, the present approach has opened up a new way to determine physical tissue properties from oblique measurements in microscopic 3D-PLI. Cortical, subcortical, and white matter regions could be characterized coherently across brain sections in terms of fiber orientation and birefringence strength. This is a prerequisite for subsequent volume-based connectivity analysis.

## Author contributions

DS: study design, algorithm implementation, simulation, processing of experimental data, evaluation, writing, discussion. SM and MS: 3D reconstruction, discussion. NS: 3D visualization. MM: provided the brain sample and anatomical interpretations. KA and TL: writing, discussion. MA: study design, discussion, writing.

### Conflict of interest statement

The authors declare that the research was conducted in the absence of any commercial or financial relationships that could be construed as a potential conflict of interest.
