# Supplementary material for: Derivation of Fiber Orientations From Oblique Views Through Human Brain Sections in 3D-Polarized Light Imaging
Source: Front Neuroanat. 2018 Sep 27;12:75. doi: 10.3389/fnana.2018.00075 (PMC6173061; doi:10.3389/fnana.2018.00075)
Supplement: Supplementary file 1 [file Presentation_1.pdf]

Supplementary Material: Derivation of fiber orientations from oblique views through human brain sections in 3D-Polarized Light Imaging

Daniel Schmitz, Sascha E.A. Muenzing, Martin Schober, Nicole Schubert, Martina Minnerop, Thomas Lippert, Katrin Amunts, and Markus Axer

*Correspondence:
Daniel Schmitz
da.schmitz@fz-juelich.de

1 CAMERA DETECTION STATISTICS

$N = 100$ images covering a broad intensity range were recorded under the same conditions to obtain a distribution of the recorded light intensities. They were analzed pixelwise for their mean $\bar{I}$ and sample variance S^2 as estimators of the expected value and variance according to

$$\bar{I} = \frac{1}{N} \sum_{j=1}^{N} I_j \tag{S1}$$

$$S^2 = \frac{1}{N-1} \sum_{j=1}^{N} (I_j - \bar{I})^2 \tag{S2}$$

with I_j denoting the intensity of the jth measurement. The 2D histogram of the obtained mean values and sample variances is given in fig. S1. We can conclude that sample variances and mean values are related by $S^2 = 3\bar{I}$.

REFERENCES

Wiese, H. (2017). *Enhancing the Signal Interpretation and Microscopical Hardware Concept of 3D Polarized Light Imaging*. Ph.D. thesis, Bergische Universitt Wuppertal

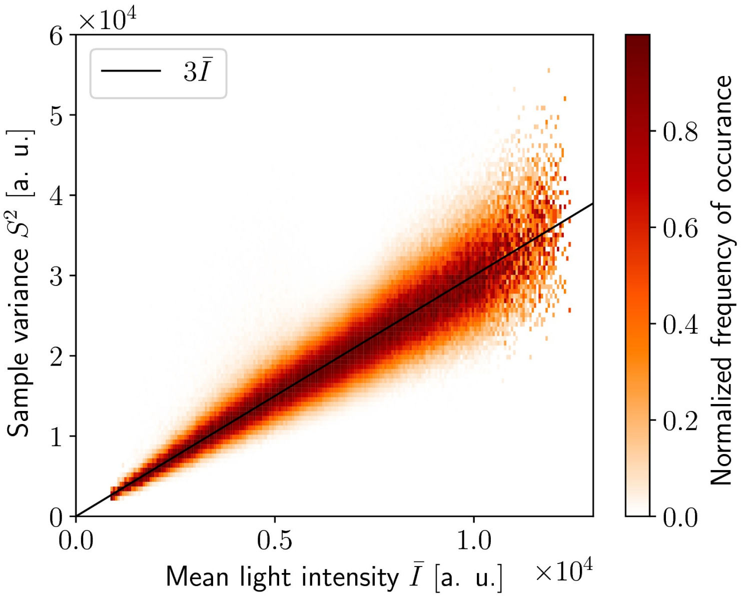

Figure S1. Normalized 2D-histogram of mean values $\bar{I}$ and sample variances S^2. The fit line shows that $S^2 = 3\bar{I}$. Modified from Wiese (2017)
